# Gödel’s modal interpretation of intuitionistic logic and its proof theory

**DOI:** 10.1007/s00605-025-02083-0

**Published:** 2025-07-08

**Authors:** Jan von Plato

**Affiliations:** University of Helsinki, Helsinki, Finland

**Keywords:** Gödel, provability logic, natural deduction, 03-03, 03F05, 03F045

## Abstract

Gödel, working within axiomatic logic, succeeded in 1933 in establishing a translation from theorems of intuitionistic propositional logic to ones of classical logic enriched with a modal provability operator. The converse correspondence was established by semantical means in 1948, and by Gödel through a syntactic translation in unpublished work of 1941. It is shown through proof analysis of formal derivations in natural deduction for modal logic that steps of indirect proof in normal derivations of translations of intuitionistic formulas are vacuous. This conservativity of classical over intuitionistic modal logic for translated formulas is the reason why Gödel’s modal translation succeeds in singling out a “provability fragment” within classical modal logic that coincides with intuitionistic logic.

## The modal interpretation

Arend Heyting’s axiomatization of the principles of intuitionistic reasoning as a formal system of proof, in his *Die formalen Regeln der intuitionistischen Logik* of 1930, was a decisive turn in the development of the constructive foundations for mathematics. It was extremely well received by Heyting’s peers, first among them Paul Bernays of Göttingen who wrote him a congratulatory letter, stating however that he had himself figured out five years earlier a proper axiomatic system for intuitionistic logic such that the addition of the law of double negation gave back the old system of classical logic. One may wonder why Bernays didn’t publish the result in 1925.^1^

Kurt Gödel, in a trial lecture for a docentship in February 1933, praises Heyting’s axiomatization in the following terms^2^

The claim that each proposition should be either true or false, completely disregarding whether one can determine the one or the other, that Brouwer holds to be senseless. Brouwer has later drawn various conclusions from his claim, in a more or less systematic way. It is only with his student Heyting that this question has been engaged with axiomatically. Namely, he has put up an axiom system for logic, more precisely the narrowest part of logic, the propositional calculus, from which the law of excluded middle cannot be derived.

He writes further that “the *tertium* does not occur at all among the logical axioms that are usually assumed, but has to be derived from them.” By usual axioms, he meant those of classical propositional logic of Russell’s *Principia Mathematica.* He further asks what should be done to have a “guarantee against the possibility that the law of excluded middle creeps in in a hidden way, through the consequences one draws from the axioms.”

Heyting’s logic was equally well received by Bernays’ student Gerhard Gentzen, who in 1932–33 formulated it in terms of natural deduction and sequent calculus, his inventions of the time. The latter formalism, in particular, led Gentzen to a mastery of the new constructive logic, with results such as the decidability of intuitionistic propositional logic, and the disjunction and existence properties.

In other quarters, namely at the mathematics institute of the University of Vienna of Hans Hahn and his bright student Gödel, Heyting’s intuitionistic logic was, as intimated, in an equally sharp focus. Hahn decided to dedicate the year 1931–32 to a *Seminar in Mathematical Logic,* with Gödel in practice responsible for the arrangements and the preparation of most of the topics to be discussed. Two themes were central in the seminar: Jacques Herbrand’s theories of arithmetic with their accompanying proofs of consistency of partial systems of arithmetic, and Heyting’s intuitionistic logic. Gödel, right after his overwhelming success with the incompleteness theorem, was able to answer most of Hahn’s questions about the nature of intuitionistic logic: A first result, published as a one-page note 1932 [5], showed that it is not a many-valued logic, say a three-valued one in which there would be in addition to the values true and false a third value, not yet decided or something. A connection between intuitionistic and modal logic was suggested by Oskar Becker in 1930 [1], an article of which Gödel [4] wrote a review published in 1931. There he writes (p. 217):

In conclusion, the author discusses, from a formal as well as a phenomenological standpoint, the connections that in his opinion obtain between modal logic and the intuitionistic logic of Brouwer and Heyting. It seems doubtful, however, that the steps here taken to deal with this problem on a formal plane will lead to success.

Next to this suggestion, the idea of a modal provability operator strikes the eye when one considers the arithmetic provability predicate ${ Bew}(p)$ encountered with Gödel’s incompleteness theorem. Last, there was a predecessor in the form of a less known Russian logician Ivan Orloff [14] whose article of 1928 Gödel had studied.^3^

Gödel’s axioms for provability in his 1933 one-page article *Eine Interpretation des intuitionistichen Aussagenkalküls* (An interpretation of the intuitionistic propositional calculus) and in the trial lecture are the same as in Orloff’s article, with Gödel writing *B* for *beweisbar* in place of Orloff’s $\mathbf \Phi$:

**Table 1**. **Orloff’s and Gödel’s provability axioms.**

1. $$B(a) \supset a$$

2. $$ B(a \supset b) \, \& \,B(a) \supset B(b)$$

3. $$B(a) \supset BB(a)$$

The rule of inference is that if *a* has been proved, *B*(*a*) can be inferred. Today Orloff’s and Gödel’s system is known as the modal logic **S4**.

Gödel proceeded by reading the propositions of intuitionistic logic in terms of provability, following Heyting’s [11] explanations of the intuitionistic connectives:

**Table 2**. **Gödel’s modal translation of 1933.**

    $$\left. \begin{array}{lr} p \vee q \hspace{20.0pt}B(p) \vee B(q) \\ p \supset q \hspace{17.0pt}B(p) \supset B(q) \\ p \wedge q \hspace{20.0pt}B(p) B(q) \\ \lnot p \hspace{27.0pt}\sim \!B(p) \end{array} \right\} \mathrm{Usual \ concepts \ at \ the \ right} $$

The “usual concepts at the right” are those of classical propositional logic, with the intuitionistic $\lnot$ and $\wedge$ replaced by $\sim$ and concatenation. At one point, Gödel uses a distinct notation throughout, $\wedge , \overset{-}{\vee }, \supset , \lnot$, and $., \vee , \rightarrow ,$ $\sim$, for the intuitionistic and classical notions, respectively. In the Gödel-Hahn seminar, he writes that “the intuitionistic basic concepts, equally designated, are presupposed to have meanings completely different from the ones known in the RW $\llbracket$Russell-Whitehead$\rrbracket$ axiom system.”^4^

The modal translation is intended so that the subformulas *p*, *q* at the right are substituted by their translations, until there are no connectives left. Thus, a formula with *m* negations and *n* other connectives gives a translation with $m + 2n$ provability-operators *B*. Gödel gives as an example the translation of $p \vee \lnot \, p$ that is $B(p) \vee B(\sim \!B(p))$.

Gödel’s article does not add the *B*-operator in the translation of conjunction, but he does it elsewhere, as in the summary of his trial lecture from which the above is drawn. The translation shown gives a useful uniformity to the structure of translated formulas.^5^

In Gödel’s times, logic was still represented in its axiomatic form, handed down from Russell through the book of Hilbert and Ackermann of 1928. Thus, his proof strategy for the theorem to be proved was wrought in such axiomatic terms: Show first that all of the axioms of intuitionistic propositional logic turn into theorems of the modal logic of provability. Next show that whenever a rule of inference is applied to premisses that turn into provable propositions of the logic of provability, also the conclusion turns into such. The net effect is that theoremhood is preserved under the translation.

Gödel conjectured in 1933 that the correspondence he had established between the theorems of intuitionistic propositional logic and those of his logic of provability is exact: He had proved that if *A* is a theorem of intuitionistic propositional logic, its translation is a theorem of the logic of provability. What was needed was a converse: If the translation of a formula *A* is a theorem of the logic of provability, *A* is a theorem of intuitionistic logic. This result was proved by semantic means in McKinsey and Tarski in 1948 [12].

Gödel added the three axioms and a rule of proof for the provability operator to “the usual logic,” i.e., classical propositional logic that clearly called for no further specification. He then showed that the translations of Heyting’s axioms are derivable: First he takes an instance of an axiom, with $B(a), B(b),\dots$ in place of the propositional variables $a,b,\dots$. Then he applies the derivable rule by which from $P \supset Q$ (assumed provable), $B(P) \supset B(Q)$ can be inferred.^6^

As an example of the proof procedure, Heyting’s axiom 5, $b \supset \!. \, a \supset b$, has the instance $B(b) \supset \!.\, B(a) \supset B(b)$. It is a tautology of “the usual logic.” By the rule, $BB(b)\,. \! \supset \!.\, B(B(a) \supset B(b))$. By modal axiom 3, the antecedent can be replaced by *B*(*b*), to obtain $B(b)\,.\! \supset \!.\, B(B(a) \supset B(b))$ that is the modal translation of axiom 5.

Gödel seems not to have thought of the possibility of an intuitionistic modal logic in which the translations of the axioms are derivable. His aim was to interpret intuitionistic logic in terms of classical logic, and it was natural in that setting to base such derivations on formulas that are tautologies of classical logic.

Gödel achieved a converse of sorts to his theorem in 1941, found in a series of four notebooks with the title *Resultate Grundlagen [8].* The translation that had eluded him in 1933 succeeded through a variant of a normal form he had learned upon meeting Gentzen in Göttingen in mid-December 1939: The usual conjunctive normal form of a formula *A* is $A_1 \, \& \, \dots \, \& \,A_k$ in which each conjunct $A_i$ has the form of a disjunction of atomic formulas and negations of atomic formulas:

$$\begin{aligned} P_1 \vee \, \dots \vee P_m \vee \lnot Q_1 \vee \, \dots \vee \,\lnot Q_n \end{aligned}$$

In particular, *A* is a tautology if and only if, for each conjunct, there are *i*, *j* such that $P_i \equiv Q_j$, as each conjunct then contains an instance of excluded middle. Gentzen introduced the form

$$  \begin{aligned} R_1\, \&  \, \dots \, \& \, R_k \supset S_1 \vee \,\dots \vee \,S_l \end{aligned}$$

This is a constructive version of a normal form that has turned out to be very useful. Gödel proves that if an implication of this form is classically derivable from given implications of this same form, it is already intuitionistically derivable. The result is a propositional form of what today is called Barr’s theorem. Gödel’s strategy is to first constructivize a formula provable in modal logic, then to gradually delete the *B*’s. The details of this proof are given in Negri and von Plato [13], together with a discussion of his contacts with McKinsey and Tarski.

Gödel’s result of 1941 was a “converse of sorts,” as it involved a constructivization of proofs within **S4**. His idea was that if the translation *T*(*A*) of an intuitionistic theorem *A* is provable in classical **S4**, there is a formula $A'$ classically equivalent to *T*(*A*) that is provable in **S4** without the use of classical principles of proof. This formula $A'$ and its proof can be translated back to intuitionistic logic.

When natural deduction is used, it becomes at once evident that the translation of an intuitionistically provable formula is provable without indirect proof in **S4**: The translation extends easily to derivations, with some added steps of elimination and introduction of the provability operator, with no instance of indirect proof visible anywhere. Thus, the proper perspective on the matter is to show that indirect proof in **S4** is conservative over the class of provable translated formulas. That is the content of our Main Lemma proved in Sect. 4.

The possibility to eliminate instances of indirect proof from derivations of translated formulas explains in a simple way why it is possible to reproduce intuitionistic logic within the classical modal system **S4**.

The aim of the pages that follow is to give a thoroughly elementary treatment of the correspondence between intuitionistic and modal logic, through the translation of normal derivations in both directions, for suitable systems of natural deduction.^7^

## Classical natural deduction

The first system of classical natural deduction for the full language of propositional and predicate logic that allows for a proof of normalization with no *ad hoc* restrictions, global proof transformations, or similar tricks, was given in von Plato and Siders [27]. Also natural deduction for the classical modal logic **S4** was covered, as an extension of a proof of normalization for intuitionistic **S4** in von Plato [22].

With classical natural deduction, the subformula property of normal derivations has to be modified so that all formulas in such derivations are, in addition to subformulas, possibly also negations of subformulas of the conclusion or the open assumptions.

In the mentioned works, negation was treated as a defined connective, by the use of a constant $\bot$ for a false proposition: $\lnot A =_{ Df} A \supset \bot$. It will turn out very useful for what follows to use a primitive notion of negation in classical natural deduction, with rules of introduction and elimination that bear a remarkable symmetry, as in (von Plato [23], p. 84)^8^:

**Table 3**. **Rules for a primitive notion of negation.**The standard rule of indirect proof comes out as a special case of rule $\lnot E$, when $\bot$ is set for *C*:The second premiss $\lnot \bot$ is a theorem by which that premiss and its derivation can be deleted, to obtain the standard rule of indirect proof in terms of the defined notion of negation.

It is also useful to set the elimination rules of natural deduction in the general form of von Plato [21], with all elimination rules following the pattern of $\vee E$:

**Table 4**.**General** *E*-**rules for** &$, \supset$.^9^The system of introduction rules and general elimination rules for &, $\vee$, and $\supset$, together with the primitive rules for negation will form the system of classical natural deduction **NK**.

In rules that close assumptions, the assumptions can occur any number of times. If an assumption is not used in an instance of a rule that can close assumptions, i.e., if it occurs 0 times, we say that it is **vacuously** discharged. The system of intuitionistic natural deduction **NI** is obtained as that special case of **NK** in which the two assumptions $\lnot A$ on top of the subderivations in rule $\lnot E$ are absent, i.e., vacuously discharged:

**Table 5**. **Ex falso as a special case of **$\lnot E$.**Definition.** *A derivation in* **NK** *is* ***normal*** *if all major premisses of* *E*-*rules are assumptions.*

As said, the proof of normalization of derivations in classical natural deduction of von Plato and Siders [27] uses the defined notion of negation. When negation is taken as a primitive, it can happen that a major premiss of an elimination is derived by rule $\lnot E$. That rule has no major premiss, but there are some obvious convertibilities with the two negation rules.

**Theorem. Normalization for NK.** *Derivations in* **NK** *convert to normal form.*

### Proof

The cases to consider, beyond the proof of normalization for **NI** with general elimination rules, concern convertibilities when the major premiss of one of &$E, \vee E$, or $\supset$ *E* is derived by $\lnot E$, and secondly cases with successive instances of rules $\lnot I$ and $\lnot E$:

1. Major premiss of &$E, \vee E$, or $\supset$ *E* derived by $\lnot E$: These cases follow the pattern of normalization of Plato and Siders [27], illustrated here for the case of conjunction. The derivation and its conversion areThe conversions for disjunction and implication are similar.

2. Rule $\lnot I$ followed by rule $\lnot E$: Consider an uppermost such occurrence. The derivation and its transformation are:As can be expected, the conversion will not make indirect proof disappear, even if there is the rule pair $\lnot I, \lnot E$. The composition formula *B* can lead to a new convertibility, but it is in a strictly smaller subderivation and therefore such cases get removed in a bounded number of steps. QED.

There are, in addition to the above obvious cases, further reductions that we take as belonging to a normal derivation:

**Lemma. Reduction of negation.** *Successive applications of the negation rules reduce to at most one such application.*

### Proof

There are four cases of combinations of $\lnot I, \lnot E$, one covered above. The three remaining reductions are:

1. Rule $\lnot I$ followed by rule $\lnot I$: The derivation and its transformation are:2. Rule $\lnot E$ followed by rule $\lnot I$: The derivation and its transformation are:3. Rule $\lnot E$ followed by rule $\lnot E$: The derivation and its transformation are:QED.

It can happen, of course, that both premisses of a $\lnot$ -rule have been derived by $\lnot$ -rules. Such cases can be resolved in two ways. An interesting case is $\lnot A$ concluded by $\lnot I$, its left premiss *B* concluded by $\lnot E$ and right premiss $\lnot B$ by $\lnot I$. If the assumption *B* of the latter is substituted by its derivation by $\lnot E$ (derivation of the left premiss of the rule that gives $\lnot A$), the result is a derivation with three instances of rule $\lnot I$. If instead the assumption $\lnot B$ in rule $\lnot E$ of the original derivation is substituted by its derivation by rule $\lnot I$ (derivation of the right premiss of the rule that gives $\lnot A$), the result is a derivation with two instances of rule $\lnot E$ and one of $\lnot I$. One of the results of permutation is a derivation in **NI**, the other a derivation in **NK**. Thus, uniqueness of normal form fails with classical natural deduction, if the notion of normality includes that there shall not be any successive negation rules.

Case 2 shows that indirect proof is not needed if the conclusion of a derivation is a negation.

**Corollary. Indirect proof reduced to one last instance in a derivation.** *Indirect proof can be permuted down with respect to all of the propositional rules, and several such rules contract into just one.*

### Proof

It is readily seen, by routine transformations, that rule $\lnot E$ permutes down with respect to all the introduction and elimination rules for &, $\vee ,$ and $\supset$. Moreover, as shown in the above lemma, consecutive instances of rule $\lnot E$ collapse into just one such instance. QED.

By the result, one indirect inference as a last rule suffices for classical propositional logic. The situation is different in predicate logic, because the variable condition in the rule of universal introduction makes it impossible to permute $\lnot E$ below it. A similar thing happens in modal logic.

The subformula property of normal derivations has to be modified so that all formulas in a normal derivation are subformulas or negations of subformulas of open assumptions or the conclusion. This follows easily for derivations with one instance of $\lnot E$ as a last rule: Instead of $\lnot E$ with conclusion *A*, conclude $\lnot \lnot A$ by rule $\lnot I$, an intuitionistic derivation with the familiar subformula property.

## Natural deduction for modal logic

The rules of the system of modal logic **S4** are, with the *E*-rule formulated in the manner of a general elimination rule:

**Table 6**. **Natural modal rules.**Rule $\square I$ has the restriction that the open assumptions $\Gamma$ on which the premiss *A* depends must be modal formulas, and the effect is the same as with rule $\forall I$, namely, that indirect proof cannot be permuted below $\square I$. The standard $\square E$ rule is obtained when $C = A$. It is also useful to have a variant of rule $\square E$ in which there are two major premisses $\square A$ and $\square B$ with the minor premiss *C* derived from *A* and *B* together, instead of two applications of the above rule. The intuitionistic and classical modal systems will be denoted by **NIS4** and **NKS4**, respectively.

Normal derivability in modal logic with general elimination rules is defined as above: Major premisses of elimination rules have to be assumptions. The normalization theorem for intuitionistic natural deduction extended by the two modal rules is proved in von Plato [22] and carries over to the classical case of the calculus **NKS4**. This was briefly noted already in von Plato and Siders [27]; to note in particular how the general rules remove the old problems of natural deduction for modal logic, such as the failure of normalization in the derivation of the theorem $\square A\, \& \,\square B \supset \square (A\, \& \,B)$ (cf. Prawitz [15], p. 76).

Before proceeding to the normalization of derivations in **NKS4**, let us check that the above natural calculus is deductively equivalent to the axiomatic calculus of Gödel. In one direction, starting from natural logic, derivations of the Orloff-Gödel axioms are easy exercises in **NKS4**, and rule $\square I$ belongs to both. The other direction, from axiomatic to natural modal logic, is based on the translation of derivations from classical natural deduction to classical axiomatic logic^10^:

Consider an uppermost instance of a modal rule in a derivation in **NKS4**. If it is $\square I$, there is a derivation in **NK** of the premiss *B* of $\square I$ from “boxed” assumptions $\Gamma = \square A_1,\dots ,\square A_n$, and we have to show that the conclusion $\square B$ of the rule is derivable in axiomatic **S4** in the form of the implication

$$\begin{aligned} \square A_1 \supset (\square A_2 \supset \dots \supset (\square A_n \supset B)\dots ) \supset \square B \end{aligned}$$

To simplify the notation, let $n=1$, so we have a derivation in **NK** of *B* from $\square A$. Rule $\supset$ $I$ gives that $\square A \supset B$ is derivable in **NK**. By the assumption, it is also derivable in axiomatic classical logic. By rule $\square I$, $\square (\square A \supset B)$ is derivable in axiomatic modal logic. By axiom 2, we can distribute $\square$ inside the implication:

$$\begin{aligned} \square \square A \supset \square B \end{aligned}$$

Axiom 3 gives $\square A \supset \square \square A$, so by the transitivity of implication, $\square A \supset \square B$.

Secondly, if the uppermost modal rule is $\square E$, we have derivations in **NK** of its major premiss $\square A$ and of the minor premiss *B* from the assumption *A*. By rule $\supset$ $I$, $A\supset B$ is derivable in **NK**. Axiom 1 gives $\square A \supset A$, so by the transitivity of implication, $\square A \supset B$ is derivable in axiomatic modal logic.

**Theorem. Normalization for NKS4.** *Derivations in* ***NKS4*** *convert to normal form.*

### Proof

The new cases to consider are when the major premiss of $\square E$ has been derived by rule $\square I$ or by an elimination rule:

1. The major premiss has been derived by $\square I$. The derivation is:A detour conversion gives:2. The major premiss has been derived by $\square E$. The derivation is:A permutative conversion gives:The height of derivation of the premiss $\square B$ of the original lower instance of $\square E$ has been diminished by one.

3. The major premiss has been derived by &$E, \vee E,$ or $\supset$ $E$. The last one is:A permutative conversion gives:The permutation of the order of the two rules is possible only with the general form of rule $\supset$ $E$. The cases for & *E* and $\vee E$ are similar.

4. The major premiss has been derived by $\lnot E$. The derivation is:The transformation is into:QED.

Whereas rule $\lnot E$ permutes down with respect to rule $\square E$, the condition in rule $\square I$, entirely analogous to the eigenvariable condition of rule $\forall I$, rules out such permutation.

**Corollary. CM-lemma.** *If in a normal derivation of a theorem in* **NKS4** *the last rule is *$\lnot E$, *the derivation of its right premiss is degenerate.*

### Proof

The derivation ends with:By normality, $\lnot C$ is not derived by rule $\lnot I$. It is not derived by an *E*-rule, because that would leave at least one assumption open. Therefore it is an assumption, with $\lnot C = \lnot A$. QED.

By the degeneracy, $C = A$, and with the right premiss left unwritten, the rule is:Here *CM* stands for *Consequentia Mirabilis,* a rule that corresponds to the axiom $(\lnot A \supset A) \supset A$. The CM-lemma holds in particular for the calculus **NK**.

## Gödel’s interpretation of the intuitionistic connectives

Let us look, in the light of the above rule system of modal logic, at Gödel’s interpretation of the intuitionistic connectives. With conjunction, provability of $A\, \& \,B$, i.e., $\square (A\, \& \,B)$, is interpreted as $\square A\, \& \,\square B$. It is easy to show that $\square A\, \& \,\square B$ and $\square (A\, \& \,B)$ are interderivable in **NIS4**, the latter asserting the provability of a classically intended conjunction.

With disjunction, it is easy to derive

$$\begin{aligned} \square A\vee \square B \vdash _\textbf{NIS4} \square (A\vee B) \end{aligned}$$

The other direction fails, even in **NKS4**, say with $\square (A\vee \lnot A)$ that is derivable in **NKS4** in contrast to $\square A\vee \square \lnot A$ that is not derivable. The underivability follows from the disjunction property for formulas $\square A\vee \square B$ in **NKS4** that will be shown in Section 6. Therefore, the intuitionistic disjunction $\square A\vee \square B$ is strictly stronger than the classical disjunction $\square (A\vee B)$.

With implication, we have again an easy derivation of

$$\begin{aligned} \square (A\supset B) \vdash _\textbf{NIS4} \square A\supset \square B \end{aligned}$$

The other direction fails, as when for *B* we put $\square A$:

$$\begin{aligned} \square A \supset \square \square A \nvdash _\textbf{NIS4} \square (A \supset \square A) \end{aligned}$$

The assumption is a theorem in **NKS4**, the claim instead gives, by $\square E$, $A\supset \square A$, which would make every assumption at once provable. Thus, the intuitionistic implication $\square A \supset \square B$ is strictly weaker than the classical $\square (A \supset B)$. The former states that the step from *A* to *B* is admissible, i.e., that from the provability of *A*, the provability of *B* follows.

Gödel’s translation appears as a direct formal counterpart to Heyting’s 1931 [11] explanation of the intuitionistic connectives in terms of conditions on provability, and that is indeed the way he motivated it. From the perspective of natural deduction, the explanations go hand in hand with the corresponding introduction rules.

## The main lemma

The translation of formulas *C* of intuitionistic logic to formulas *T*(*C*) of modal logic is defined by cases. In the definition, *A*, *B* denote arbitrary formulas and *P* an atomic formula:

**Table 7**. **The modal translation.**

1. $$T(P) = \square P$$

2. $$  T(A\, \& \,B) = \square (T(A))\, \& \,\square (T(B))$$

3. $$T(A\vee B) = \square (T(A))\vee \square (T(B))$$

4. $$T(A\hspace{-0.5pt}\supset \! B) = \square (T(A))\hspace{-0.5pt}\supset \! \square (T(B))$$

5. $$T(\lnot A) \hspace{13.0pt}= \lnot \square (T(A))$$

Let formulas with one of &, $\vee , \supset , \lnot$ as the main connective be called **I***-formulas* and ones with $\square$ as the main connective **S***-formulas.* The translation as defined above gives the following uniform structure to sequences of immediate subformulas of the translation *T*(*C*) of a given formula *C*:

Assuming *C* has some logical structure as in $A \circ B$ or $\lnot A$, we have the translations $\square (A )\circ \square (B)$ and $\lnot \square (A)$, with the translations continuing from the immediate subformulas of $A \circ B$ and $\lnot A$, and call the final result a **T**-formula. The translation *T*(*C*) is an **I**-formula and its immediate subformulas are **S**-formulas. The immediate subformulas of these latter are in turn **I**-formulas, followed by **S**-formulas, etc. In the end, Gödel’s translation procedure produces an alternating sequence of immediate subformulas, an **I**-formula, followed by an **S**-formula, an **I**-formula, etc, until atomic formulas $P,Q,R,\dots$ are reached that become the **S**-formulas $\square P,\square Q,\square R,\dots$. Here is an example:

$$\begin{aligned} T((A \supset \lnot B) \supset (B \supset \lnot A)) = \square (\square A \supset \square \lnot \square B) \supset \square (\square B \supset \square \lnot \square A) \end{aligned}$$

Normal derivations in natural deduction with general elimination rules, including the modal extension, have a very clear subformula structure. With Gentzen’s rules in the $\vee ,\exists$-free fragment, the *E*-rules move from the premisses to an immediate deductive consequence that is at the same time also an immediate subformula of a premiss. With the general *E*-rules, the subformula structure is carried along in *threads* that start from an outermost major premiss of an *E*-rule, one that is not subordinate to any previous *E*-rule. It then moves to the immediate components of the principal formula of the *E*-rule, which can again be major premisses of an *E*-rule, etc, until there appear the components of the innermost major premiss of an *E*-rule that are not anymore analyzed into components by a further *E*-rule. Now begins the part of the thread in which there is a succession of *I*-rules, with duplications at places in which the conclusion of an *E*-rule is drawn.

Each subderivation of a minor premiss of rule $\supset$ $E$ inherits the above global structure.

With formulas of intuitionistic logic translated into ones in **S4**, the curious subformula structure along threads is immediately reflected in the structure of normal derivations in the intuitionistic and classical modal calculi: **NI**-rules must alternate with $\square$-rules. The assumptions in steps of indirect proof can have negated **I**-formulas, the only exceptions to the alternating **I-S** subformula structure in derivations.

After these preliminaries, let us first give a proof of Gödel’s 1933 result, through a translation of derivations in **NI** to ones in **NIS4**: First translate all the formulas in the derivation, then insert modal rules as needed between each two instances of rules of **NI**. Major premisses of *E*-rules in normal derivations can be nested, say the premiss $(A \supset B)\, \& \,A$ of rule &*E* and the premisses $A\supset B$ and *A* in rule $\supset$ $E$. The translation turns the major premiss of the first rule into $\square (\square A\! \supset \! \square B)  \&  \square A$, with the conjuncts $\square (\square A\! \supset \! \square B)$ and $\square A$. Therefore rule $\supset$ $E$ has to be preceded by an instance of rule $\square E$, to recover the premisses of $\supset$ $E$, this time in the form $\square A \supset \square B$ and $\square A$. Dually, to reproduce the **I-S** subformula structure, if the conclusion of an introduction rule other than $\square I$ is a premiss in another introduction rule other than $\square I$, rule $\square I$ has to be applied after the introduction rule.

**Theorem. Modal translation of intuitionistic derivations.** *If* *C* *is a derivable formula in* ***NI***, *its modal translation* *T*(*C*) *is derivable in* ***NIS4***.

### Proof

We define a translation from intuitionistic derivations to derivations in **NIS4**. In what follows, to abbreviate the notation, the formulas *A*, *B*, *C* in the translated rules stand for *T*(*A*), *T*(*B*), *T*(*C*).

1. An assumption *A* becomes the assumption *T*(*A*).

2. Rules for conjunction. For &*I*, the step of derivation and its translation is:Rule $\square I$ is applied if the conclusion $A\, \& \,B$ of the given derivation is a premiss in an introduction rule. Otherwise it is not applied. The reason why rule $\square I$ can be applied is somewhat intricate. Assumptions have been turned into **I**-formulas. By the alternating **I-S**-structure, **I**-formulas cannot be premisses in any *I*-rules except $\square I$, else they are major premisses in elimination rules. The immediate subformulas of these assumptions are **S**-formulas and they appear as assumptions in the derivations of minor premisses of *E*-rules. – An easy way to see that the condition in rule $\square I$ is respected is to apply Prawitz’ “modal bar” criterion: If from the premiss of rule $\square I$ up, an **S**-formula is encountered along each branch, the rule can be applied. As can be seen, this is the case with the derivation at the right.

For & *E*, we have:The two-premiss variant of rule $\square E$ has been used. When the major premiss of an *E*-rule is translated, its immediate components are **S**-formulas. The intuitionistic step can be carried through only when the $\square$’s have been eliminated by an instance of rule $\square E$. The derivation of the minor premiss of rule $\square E$ will also get translated, according to the rules found in it. If the conclusion *C* of &*E* is a premiss in an introduction rule, rule $\square I$ is applied after rule $\square E$.

3. Rules for disjunction. This case is analogous to 2.

4. Rules for implication. For $\supset$ $I$, we have, with the same clause on rule $\square I$ as in 2:For $\supset E$, we have:If the conclusion *C* is a premiss in an introduction rule, rule $\square I$ is applied before rule $\supset$ $E$.

5. Rules for negation. With $\lnot I$, we have, with the same clause on rule $\square I$ as in 2:For $\lnot E$, we have:If $\lnot E$ is the last rule of the whole derivation, its conclusion is *A* instead of $\square A$. QED.

The result is an improvement on Gödel’s. Namely, as was to be expected, classical logic does not enter at all in normal derivations of translations of theorems of intuitionistic logic. Here is an example derivation in **NI**:The translation gives: We shall now exploit the alternating **I-S** subformula structure of translated formulas. That structure acts like a “modal straitjacket,” imposing constraints on the structure of normal derivations, to the effect that the rule of indirect proof cannot be applied at all in normal derivations of **T**-formulas in **NKS4**:

**Main Lemma. Conservativity of NKS4 over NIS4 for T-formulas.** *If the translation* *T*(*C*) *of an intuitionistic formula* *C* *is derivable in* **NKS4**, *it is already derivable in* **NIS4**.

### Proof

Consider an uppermost step of $\lnot E$ in a normal derivation in **NKS4**:In the derivation of the right premiss $\lnot B$ of rule $\lnot E$, assume the assumption $\lnot A$ is not vacuous, the vacuous case postponed to the end. It is not a premiss in any *I*-rule: Rule $\square I$ is excluded by its restriction and the other *I*-rules would lead to formulas that don’t have the alternating **I-S** subformula structure. Therefore $\lnot A$ is a premiss in rule $\lnot I$. The other premiss is not $\lnot \lnot A$ because then the conclusion of $\lnot E$ would be a negative formula for which no indirect proof is needed. Therefore the other premiss is *A*, derived from some assumption *C*, and the conclusion of $\lnot I$ is $\lnot C$, as in$\lnot C$ cannot be a minor premiss in rule $\supset$ $E$, because the immediate subformulas of implications must be **S**-formulas. $\lnot C$ is not a premiss in rule $\lnot I$ as that would give two successive instances of $\lnot I$. Therefore we must have $\lnot B = \lnot A$, then also $B = A$ and the derivation is:If *A* is the conclusion of the whole derivation, it is an **I**-formula, and the same if it is a premiss in rule $\square I$. Then, if *A* is a negation, the step of indirect inference is not needed. Therefore *A* is equal to one of $\square D\, \& \,\square E, \square D \vee \square E$, or $\square D \supset \square E$, and *A* is derived by &$I, \vee I$, or $\supset I$. In each of these rules, the premisses contain one of $\square D$ or $\square E$, but in the presence of the negative assumption $\lnot A$, these cannot have been derived by rule $\square I$. Therefore the assumption $\lnot A$ is not used in the derivation of the premiss *A* and the step of $\lnot E$ can be left out to obtain a derivation of *A* in **NIS4**.

Finally, the case in which the assumption $\lnot A$ is vacuous in the derivation of the right premiss of $\lnot E$, in the first proof figure above: Here *B* must have the form $\square D$. Its derivation in the left premiss of $\lnot E$ must be by rule $\square I$ and that can be, just as before, only if the assumption $\lnot A$ was absent in the derivation of the left premiss. *A* then follows by *ex falso* in **NIS4**.QED.

As can be seen, the *CM*-lemma holds also for derivations of theorems in **NKS4**.

## The consequences

Results about the relations between intuitionistic and modal logic are now mostly easy corollaries to the Main Lemma:

**Corollary 1. Translation of derivations from NKS4 to intuitionistic logic.** Consider a translated formula *T*(*C*) that is derivable in **NKS4**. It is then derivable in **NIS4**, and such derivations, when normal, have a very clear structure of alternating logical rules of **NI** and modal rules of **NIS4**. When the modal rules and boxes in the formulas are successively deleted from a derivation of *T*(*C*), there remains an intuitionistic derivation of formula *C*, with the same structure of the **NI**-rules. An example will illustrate the translation: The intuitionistic theorem $(A \supset \lnot B) \supset (B \supset \lnot A)$ gets translated into

$$\begin{aligned} \square (\square A \supset \square \lnot \square B) \supset \square (\square B \supset \square \lnot \square A) \end{aligned}$$

Here is a normal derivation in **NIS4**:The translation into derivations in **NI** consists in a simple rewriting: Start from uppermost formulas, delete all the modal operators and instances of the modal rules, and write just the rule instances of **NI**. With this procedure, the above derivation becomes:**Corollary 2. Exact correspondence between intuitionistic and modal logic.** *If* *C* *is a purely classical tautology,* *T*(*C*) *is not provable in* **NKS4**.

### Proof

Assume *T*(*C*) to be provable in **NKS4**. By the Main Lemma, it is then provable in **NIS4,** and by the translation of derivations, *C* is provable in **NI**, contrary to assumption. QED.

**Corollary 3. Disjunction property.** Gödel mentions in his 1933 article on the provability interpretation of intuitionistic logic the disjunction property of theorems of the form $\square A \vee \square B$, as a property suggested by the intuitionistic meaning of disjunction. How the property is to be proved is not revealed. In his trial lecture of the same year, he says about the disjunction property for intuitionistic logic that “the proof for this is, though, rather complicated.”^11^

Assuming Gödel in 1933 had a proof of the disjunction property for theorems of the form $\square A \vee \square B$, that for disjunctions in intuitionistic logic would follow once a translation from **S4** to intuitionistic logic is achieved. Gödel gives such a translation in the *Resultate Grundlagen* of 1940–42, theorems 63 and 64, but he in no way indicates that the disjunction property for intuitionistic logic would be an immediate consequence.

The disjunction property for theorems of the form $\square A \vee \square B$ is by the normalizability of derivations immediate for **NIS4,** the last rule of necessity being $\vee I$. For **NKS4**, in the case of a **T**-formula, the disjunction property follows because indirect proof need not be considered.

A normal derivation of a theorem of the form $\square A \vee \square B$ in **NKS4** ends with $\vee I$ or $\lnot E$, the former giving at once the disjunction property in question. With $\lnot E$ as the last rule, we have by the CM-lemma the derivation:The premiss $\square A \vee \square B$ of $\lnot E$ must have been derived by $\vee I$, say from $\square A$. $\square A$, in turn, cannot have been derived by an *E*-rule because that would leave an open assumption. The *I*-rule that gives $\square A$ is $\square I$, but it is applicable only if the assumption $\lnot (\square A \vee \square B)$ in rule $\lnot E$ is vacuous. Therefore the latter rule can be left out, with $\vee I$ as the last rule.

**Corollary 4. Decidability.** From the normalization of derivations in **NIS4** and **NKS4** follows decidability of derivability and the subformula property, with the additional clause for the latter that also negations of subformulas be counted: There is a bounded number of formulas that can occur in any normal derivation of a formula from given assumptions, and therefore a bounded number of loop-free derivations. This observation follows at once from normalization for **NIS4** [22] and for **NKS4** [27], without considering the Main Lemma.

## What does the provability interpretation prove?

Gödel’s aim with the modal interpretation seems to have been simply to understand what Heyting meant with his codification of the principles of intuitionistic reasoning. Gödel’s sources here are Heyting’s 1930 article [10] article that gives the formal system, and Heyting’s address at the Königsberg meeting in September 1930 that Gödel attended in person, with publication in 1931. In the trial lecture, Gödel explains the intuitionistic meaning of disjunction through the disjunction property, then adds (page 1 of Gödel’s text):

It is precisely this difference in the meaning of the basic concepts that explains why many theorems of the usual logic don’t hold in the Brouwerian one.

Gödel’s provability interpretation makes indeed at once clear why some classical logical laws fail in terms of the interpretation. His first example is the translation of $A\vee \lnot A$, which is $\square A \vee \square \lnot \square A$. In the trial lecture, he gives the reading: “*A* is either provable or demonstrably unprovable.” By the disjunction property, if $\square A \vee \square \lnot \square A$ is derivable in **NKS4**, one of $\square A$ and $\square \lnot \square A$ is derivable, but there clearly are no such derivations in **NKS4**. Another example is the law of double negation that becomes translated as $\square \lnot \square \lnot \square A \supset \square A$. Gödel explains this as:

The left side states that one can show that *A* is not demonstrably unprovable, and the right side that *A* is provable.

Gödel’s incompleteness article contains an undecidable proposition *A*, one for which neither *A* nor non-*A* is provable. The first known ideas about incompleteness are recorded in a discussion with Rudolf Carnap in December 1929, recorded in Carnap’s diaries^12^

5 3/4 – 8 1/2 Gödel. About the inexhaustibility of mathematics (see separate sheet). He has been provoked to these thoughts by Brouwer’s talk in Vienna; Mathematics is not formalizable without residue. He seems to be right.

Following Heyting’s explanation of intuitionistic logic, as in his Königsberg address of 1930 ([11]), Gödel found out something that must have deeply impressed him, namely that Brouwer by his denial of the universal validity of the law of excluded middle had expressed on a general level what he had just proved in a special case with his incompleteness theorem: The existence of true but unprovable propositions in any formal system of proof that contains arithmetic. Indeed, with a Gödel-sentence *G* in place of *A* in the law of excluded middle, the provability interpretation states that either *G* is provable or it is provable that *G* is not provable, but neither is the case within the system of arithmetic.

The provability interpretation points to the essential differences in the notions of disjunction and implication in intuitionistic and classical logic. Gödel’s original table of translation as reproduced above has the words: “usual concepts at right.” These concepts are the connectives of classical logic, ones Gödel tried to keep even notationally distinct from those of intuitionistic logic. Thus, in Gödel’s eyes, intuitionistic formulas became interpreted as a fragment of classical formulas augmented by the provability operator as dictated by the translation. For this correspondence to be exact, it should be the case that if a formula *C* is only classically provable, its translation *T*(*C*) should not be provable in **NKS4** – or, for Gödel, the equivalent in axiomatic logic. This, precisely, is stated by Corollary 2 of the Main Lemma.

In his notes for the trial lecture of February 1933, Gödel shows that the translations of Heyting’s axioms of intuitionistic propositional logic are tautologies and that such tautologicity is maintained by the rules of inference. He clearly did not pause to reflect on the question whether any genuinely classical principles are required in this validation of the translation. Our proof-theoretic proof of Gödel’s result shows that this is not the case.

The difference in intuitionistic and classical concepts was illustrated in Section 4. In particular, intuitionistic disjunction is interpreted, within the classical calculus **NKS4**, by the formula $\square A \vee \square B$, an interpretation justified by the disjunction property for such formulas in **NKS4**.

Similarly, we have for the classical implication $\square (A \supset B)$ within **NKS4**:

$$\begin{aligned} \square (A\supset B)\vdash _\textbf{NKS4} \lnot A \vee B \end{aligned}$$

With intuitionistic implication interpreted in **NKS4** as $\square A \supset \square B$, we have:

$$\begin{aligned} \square A\supset \square B \nvdash _\textbf{NKS4} \lnot A \vee B \end{aligned}$$

This underivability result can be shown through a failed proof search in sequent calculus. The situation remains the same if $\lnot A \vee B$ is replaced by its translation $\square \lnot \square A \vee \square B$.

The possibility to eliminate instances of indirect proof in derivations of translated intuitionistic formulas shows in concrete terms how Gödel’s modal translation succeeds in singling out a fragment of classical **S4** that is isomorphic to intuitionistic logic. The isomorphism is not only one of theorems, but of normal derivations in the two systems of natural deduction **NI** and **NIS4**.

The translation of formulas and derivations is easily extended to the quantifiers, the classical and intuitionistic notions being $\square \forall xA(x)$ and $\forall x \square A(x)$, and $\square \exists xA(x)$ and $\exists x \square A(x)$, respectively. An easy derivation shows that $\forall x \square A(x)$ follows from $\square \forall xA(x)$, but the other direction fails because of the condition in rule $\square I$, by which classical universality is stronger than its intuitionistic counterpart. Intuitionistic existence, in turn, is stronger than classical existence; $\square \exists xA(x)$ follows from $\exists x \square A(x)$, but the derivation of $\exists x \square A(x)$ from $\square \exists xA(x)$ fails because of the condition in rule $\square I$.

At the time when Gödel worked on the provability interpretation, Gentzen was perfecting his sequent calculus. One of his results of the spring of 1933 was the disjunction property for intuitionistic predicate logic, another the decidability of intuitionistic propositional logic. Gödel never fully understood Gentzen’s method which was to formulate logical calculi so that all derivations convert to a normal or cut-free form, with a subformula property for such converted derivations as a corollary. Thus, in 1941 in the *Resultate Grundlagen,* Gödel uses over ten pages for a very complicated proof of the decidability of the implicational fragment of intuitionistic propositional logic, one that follows at once from Gentzen’s subformula property of cut-free derivations in a sequent calculus for intuitionistic propositional logic.

Gödel’s modality ${\mathfrak {B}}$ in his 1933 article was intended as provability. If it is read in today’s standard terms of modal logic, as necessity, the question of the dual notion of possibility arises. There seems to be no unanimity on the properties of this notion, however. A weakest notion of possibility, notation $\lozenge A$, and one that in the Gödelian context would correspond to consistency, would be to define it as stating that the contrary is not necessary, as in:

$$\begin{aligned} \lozenge A \equiv \lnot \square \lnot A \end{aligned}$$

Gödel would perhaps have expressed this as: Anything that is not demonstrably unprovable is consistent. With this compelling idea, **NIS4** can be maintained without changes as the intuitionistic version of **S4**.

## Remarks on previous literature

Anne Troelstra [20] gives in *Basic Proof Theory* (1996) a classical multisuccedent sequent system for **S4** and proves a cut elimination theorem. This is exploited in a proof of the faithfulness of the modal embedding of intuitionistic logic. He notes that if the translation of a formula is derivable in the sequent calculus for **S4**, a cut-free derivation uses only intuitionistically valid instances of the sequent rules, by which faithfulness follows. Nothing more is made of this profound observation. His comments to Gödel’s [6] article, in volume I of Gödel’s *Collected Works* (p. 299), emphasize instead that “**S4** is a system based on *classical* logic.”

Ulrich Kohlenbach pointed out that Schütte’s [18] little book of 1968, *Vollständige Systeme modaler und intuitionistischer Logik* contains a syntactic proof of faithfulness of the modal embedding. The intuitionistic calculus, section 13, is similar to the sequent calculus in Troelstra. The modal system for the embedding and its inverse is instead based on Schütte’s pet idea, what are called positive and negative parts of formulas. The effect is the same as with his book *Beweistheorie,* namely, that his proof remained largely unread. Moreover, not a word is wasted on what the significance of the result could be in foundational research.

There is an old paper by Prawitz and Malmnäs [16] in which the result is announced that indirect proof can be eliminated from derivations of translated formulas in a system of natural deduction for **S4**. The suggested proof is, however, formulated in very general terms, without the intricate combinatorial details that lead to the result.

Finally, I wish to thank Sara Negri who in her presentation “Gödel, Barr’s theorem, and the logic of provability: account of an anticipation” at the conference in honour of Gabriel Sandu in Helsinki, Finland, November 28, 2022, summarized the previous literature and then showed the faithfulness of Gödel’s embedding through the machinery of labelled sequent calculus (see Dyckhoff and Negri [3]). That approach leads to vast new results in logic, whereas here the aim has been to show that the correspondence between intuitionistic and modal logic can be established with very elementary means.

## Appendix: Basic notions of Gentzen’s natural deduction

Gerhard Gentzen’s doctoral thesis of 1933, *Untersuchungen über das logische Schliessen,* introduced the two main systems of proof of today, namely natural deduction and sequent calculus. The former arose from an analysis of how proofs in mathematics are actually carried out. Theorems typically state that a thing follows under some given assumptions. Two kinds of steps are possible: First, the assumptions can be analyzed into components by *elimination rules,* say, to derive from a conjunction $A\, \& \,B$ the conjunct *A* or to take from a universal $\forall xA(x)$ an instance *A*(*c*). Secondly, the thing to be proved can be reduced into simpler subtasks in *introduction rules,* as when the goal of proof $A\, \& \,B$ is replaced by the simpler goals *A* and *B* or the goal $\forall xA(x)$ replaced by the task to prove *A*(*x*) for an *arbitrary* *x*.

Gentzen’s system of natural deduction has the following rules for conjunction, disjunction, and implication:

Square brackets indicate temporary assumptions made during a search for a derivation, with the open assumption *closed* when the conclusion is drawn, as indicated by the numerical labels that show which temporary assumption is closed where. In elimination rules, the premiss with the connective is *the major premiss* and possible other premisses are *minor premisses.* The number of assumptions closed in a step of inference can vary. If it is the limiting case 0, i.e., the assumption was in fact not used, we say that the assumption is *vacuously discharged,* and if it is $> 1$, it is *multiply discharged.*

We see from the rules that, e.g., an implication $A\supset B$ to be proved is replaced by *B* to be proved from the assumption *A*. Note that the elimination rule for disjunction has two cases, and if *C* follows from both, it follows from $A\vee B$.

Formally, the class of *derivations* is defined inductively by two clauses: 1. Any formula *A* is a derivation of *A* from the open assumption *A*. 2. Given derivations of the premisses of a rule from some open assumptions, application of the rule gives a derivation of its conclusion from the same open assumptions minus any closed assumptions. Thus, derivations are two-dimensional tree-like combinatorial objects, with the following remarkable property that we can describe as the *exact representation of the deductive dependences* in a proof: A formula in a derivation tree depends on precisely those open assumptions that appear as leaves of the subtree determined by the formula. Here is a simple example derivation in which the nested conditions *A* and *B* in $A \supset (B \supset C)$ are brought together:

Gentzen’s terminology is suggestive of how introduction rules lead to a formula and elimination rules dispose of that formula. Thus, there can appear in the middle of a derivation patterns such as for the above three:

In each case, a derivation can be constructed by *composition* of parts of the given derivation in which the formula with the connective is removed:

Gentzen said that these derivations don’t make any “detours” and thus, the above are today called *detour conversions.*

It is possible that the above compositions create new detours: Say, maybe the minor premiss *C* in the second was derived by some *I*-rule and then became, in the conclusion of the rule, a major premiss in an *E*-rule. To deal with this case, Gentzen took into use the idea of a *permutative conversion:* The *E*-rule instance found after $\vee E$ is moved to above the latter, and it becomes one of the above three detour convertibilities. A similar thing can happen with the other two conversions, but note that the possible new detours in these derivations are on formulas strictly shorter than the original ones. Therefore the conversion of detours stops at some point.

The main result about natural deduction is Gentzen’s “normalization theorem” by which all derivations convert into a form in which there are no detour or permutation convertibilities left. Such derivations are said to be in *normal form.* Their main property is that all formulas in such derivations are parts of the open assumptions or the conclusion, what is called the *subformula property.*

## Footnotes

1 Bernays’ letter to Heyting is reproduced as an appendix in Troelstra [19].

2 Gödel’s lecture is found in the seminar proceedings [9]).

3 Details on Orloff’s article are found in Negri and von Plato [13]. Gödel wrote, apparently in the fall of 1932, a summary of Orloff’s article. Whether he had seen it before his own work remains undecided. The summary contains (von Plato [25], p. 132): $\rightarrow$ should be interpreted as: follows logically. Further, a theory about *Bew* that rests on three axioms: $\Phi a \rightarrow a, \hspace{3.0pt}\Phi a \rightarrow \Phi \Phi a, \hspace{3.0pt}\Phi (a \rightarrow b) \rightarrow \Phi a \rightarrow \Phi b$.

4 The former is in frame 913 of reel 24, the latter in the seminar of 18 January 1932.

5 The translation is found on frame 916 of reel 24 of the Gödel microfilm collection.

6 These proofs, with no words of explanation, are found in the Gödel papers among materials included in a folder “Hahn logic seminar 1931/32.” They are very carefully written on pages numbered I–VIII, Gödel microfilms, reel 24, frames 905–912. The Gödel-Hahn seminar presents some of them with explanations, tenth seminar of 25 January, 1932. Gödel’s one-page article of 1933 says nothing at all about these proofs.

7 See the appendix for a recollection of the basic notions of natural deduction.

8 These rules were suggested already by Gentzen himself in unpublished notes from 1932. See von Plato [24], p. 113.

9 The standard rules are the degenerate cases:

10 Cf. (von Plato [23], p. 55 ff.) for the translation, modulo the new primitive rules for negation.

11 Gödel’s trial lecture, *Über den intuitionistischen Aussagenkalkül* (On the intuitionistic propositional calculus) in Gödel and Hahn [9]. The quote is from Gödel’s page 4.

12 Now published as Carnap [2]. It turned out in August 2022 that there is a one-page typewritten explanation of Gö:del’s ideas with the same date as Carnap’s diary remark, placed with the Gödel-Carnap correspondence in Princeton. The text is found in *Portrait of Young Gödel,* p. 111.
